# Supplementary material for: eNOS-NO-induced small blood vessel relaxation requires EHD2-dependent caveolae stabilization
Source: PLoS One. 2019 Oct 10;14(10):e0223620. doi: 10.1371/journal.pone.0223620 (PMC6786623; doi:10.1371/journal.pone.0223620)
Supplement: S1 Table — (DOCX) [file pone.0223620.s001.docx]

**Supporting information**

**S1 Fig. EHD2 antibody staining in EHD2 del/+ vs. EHD2 del/del arteries.** Cryosections of small vessels (A, C) or aorta (B, D) obtained from EHD2 del/+ or EHD2 del/del mice were stained against EHD2 and eNOS and visualized by confocal microscopy. Please note that the resolution obtained by this analysis was not sufficient to distinguish the cellular membrane compartments.

**S2 Fig. Angiogenesis in EHD2 del/+ and del/del mesenteric artery pieces.** (A) EHD2 del/+ mice were bred with EHD2 del/del mice, offsprings were genotyped and the gender was analyzed. 27-35 breeding events were investigated. (B) EHD2 del/+ and del/del mesenteric arteries were dissected and small pieces were cultivated on matrigel. Scale bar 40 µm.

**S3 Fig. Functional analysis of EHD2 del/+ and del/del mesenteric arteries (related to Fig 2).** (A) Example traces of active force measurements in EHD2 del/+ and del/del mesenteric arteries illustrated the same vessel contraction ability and a total recovery of contraction after KCl wash out after 30 min. Please note that in most of the experiments, we did not wait until full recovery to accelerate the experiment and therefore increase the number of experiments with mesenteric arteries pieces of the same animal (typically 7-10 per day and animal). (B) Muscarinic acetylcholine receptor M3 staining in mesenteric artery cryostat sections revealed no differences in M3 protein level in EHD2 lacking mice compared to control mice. The M3 staining intensity was determined by ImageJ/Fiji, in which 350 µm^2^ sub-regions of the vessels were analyzed (n(del/+) = 51/3, n(del/del) = 46/3, graph illustrates each replicate with mean +/- SE, FI – fluorescence intensity).

**S4 Fig. STED imaging of eNOS and Cav1 in EHD2 del/+ and del/del mesenteric arteries (related to Fig 3).** EHD2 del/+ and del/del mesenteric arteries were cryosectioned, stained against eNOS (cyan) and Cav1 (magenta) and the distribution investigated by STED microscopy. Scale bar 4 µm.

**S5 Fig. EHD2 siRNA knockdown in HUVEC (related to Fig 4 and 5).** (A) HUVECs were treated with EHD2 siRNA or non-sense siRNA (Negt. CTRL) and stained against EHD2 (red) and Cav1 (green). (B) EHD2 staining intensity revealed a significantly reduction for EHD2 siRNA#1 and #2 (n(Negt. CTRL) = 7/3, n(siRNA#1) = 10/3; n(siRNA#2) = 13/3, box plots represent mean with whiskers from min to max, * P<0.05; *** P<0.0001). (C) Western Blot analysis of HUVECs treated with EHD2 siRNA or non-sense siRNA.

**S6 Fig. Model of EHD2 function in endothelial cells.** Loss of EHD2 results in detachment of caveolae from the plasma membrane (see our EM results, Fig 1 and 4). Consequently, eNOS does not localize to the plasma membrane (as illustrated here by STED imaging, see Fig 3, 4 and S2 Fig). In addition, store operated Ca^2+^ channels (SOCE) known to localize within caveolar membrane domains are not active resulting in reduced Ca^2+^ entry after acetylcholine treatment, as shown in Ca^2+^ recordings in EHD2 siRNA treated HUVECs (Fig 5). Due to the reduced cytosolic Ca^2+^ concentration, CaM is not activated and can therefore not bind to eNOS resulting in reduced eNOS activity, as shown by decreased phosphorylation levels of Ser1177-eNOS, Fig 5, and, consequently, reduced NO concentrations, as illustrated by DAF staining in EHD2 del/del mesenteric arteries and HUVECs treated with EHD2 siRNA, Fig 3 and Fig 5). Due to the reduced generation of NO, vasodilation in small vessels of EHD2 del/del mice is impaired (see active force measurements in Fig 2).

**S1 Table. Echocardiography of 20 weeks old EHD2 del/+ and del/del mice before and after 2 weeks running wheel training.**

**S7-9 Fig. Raw images of Western Blots files.**

Within the paper, supporting information files, in a public repository:

DOI 10.17605/OSF.IO/RBVJZ

**S1 Table. Echocardiography of 20 weeks old EHD2 del/+ and del/del mice before and after 2 weeks running wheel training.**

|  | **Before training** | | **After training** | |
| --- | --- | --- | --- | --- |
|  | **EHD2 del/+** | **EHD2 del/del** | **EHD2 del/+** | **EHD2 del/del** |
| LV posterior wall thickness, dia [mm] | 0.7±0.1 | 0.8±0.1 | 0.7±0.1 | 0.8±0.1 |
| LV posterior wall thickness, sys [mm] | 0.9±0.1 | 1.1±0.1 | 1.0±0.1 | 1.0±0.1 |
| LV mass, uncorrected [mg] | 134±5 | 150±10 | 144±8 | 144±8 |
| Fraction shortening [%] | 21±1 | 21±1 | 21±1 | 24±1 |
| Ejection fraction [%] | 42±2 | 43±2 | 42±2 | 47±2 |
| Stroke volume [µl] | 32±2 | 29±1 | 28±1 | 29±1 |
| Cardiac output [ml/min] | 14±1 | 14±1 | 12±1 | 13±1 |
| Heart rate [bpm] | 440±20 | 480±10 | 450±20 | 450±20 |
