# Supplementary figures and images for: eNOS-NO-induced small blood vessel relaxation requires EHD2-dependent caveolae stabilization
Source: PLoS One. 2019 Oct 10;14(10):e0223620. doi: 10.1371/journal.pone.0223620 (PMC6786623; doi:10.1371/journal.pone.0223620)

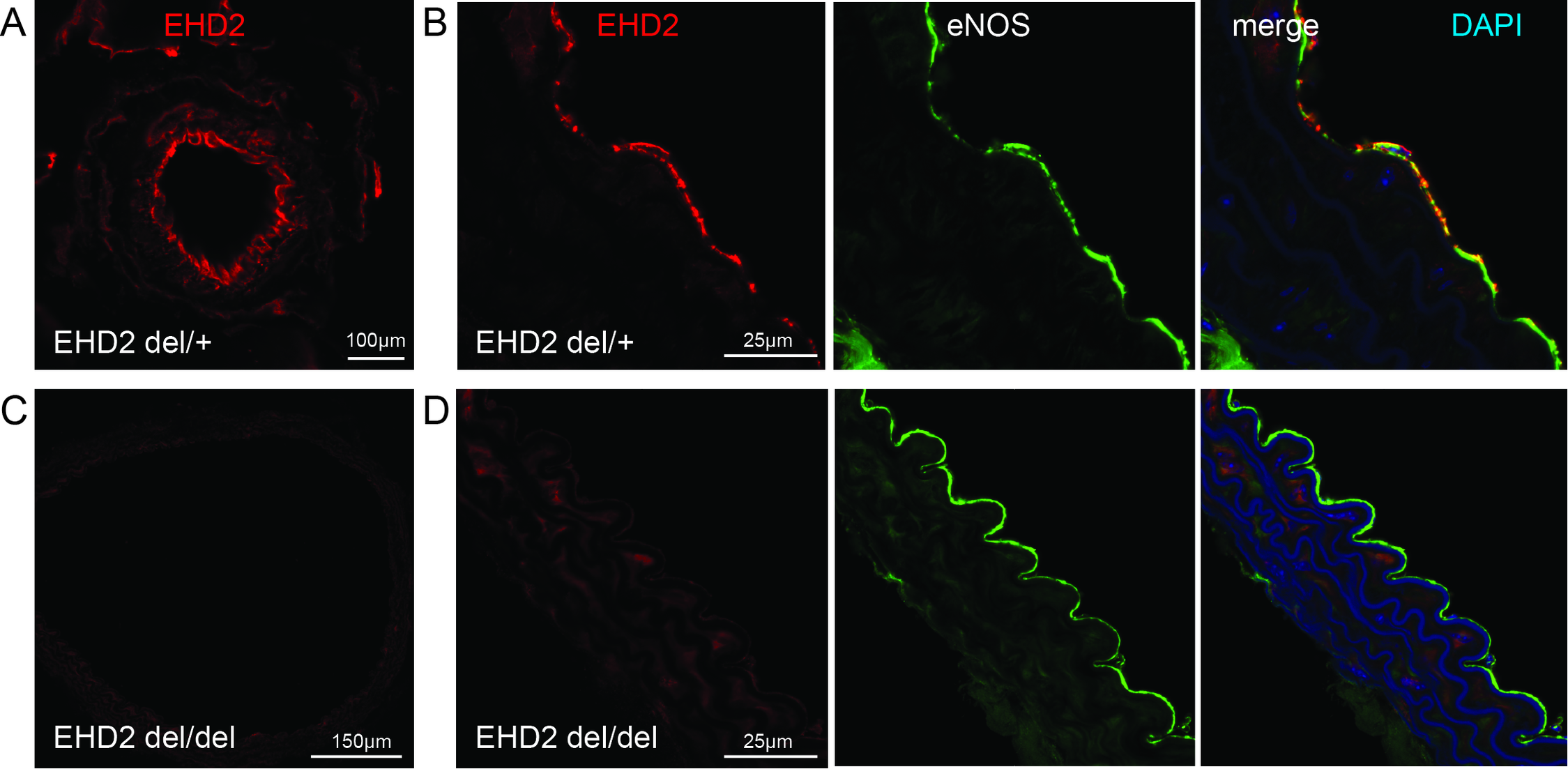

Supplement: S1 Fig — (TIF) [file pone.0223620.s002.tif]

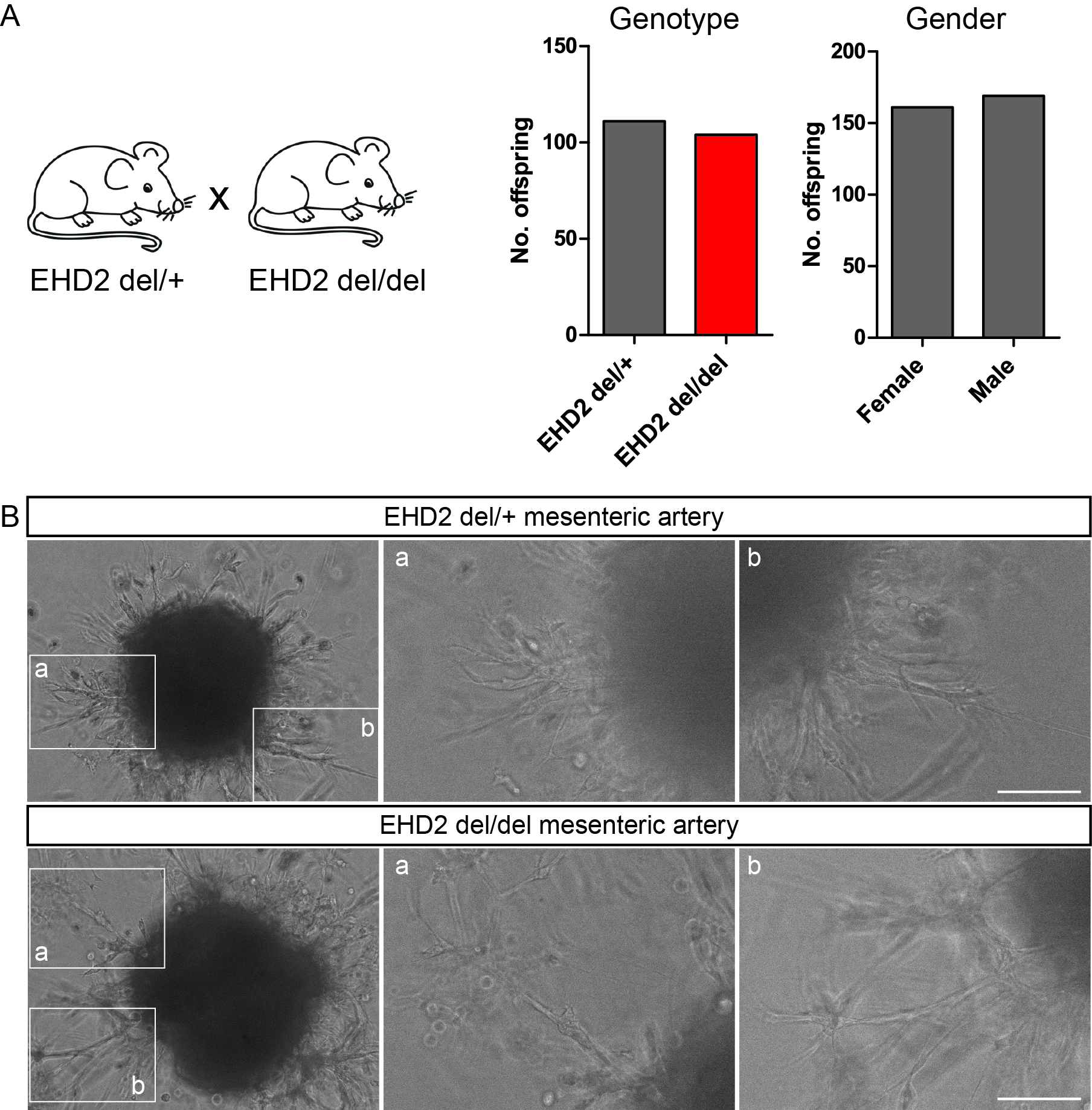

Supplement: S2 Fig — (TIF) [file pone.0223620.s003.tif]

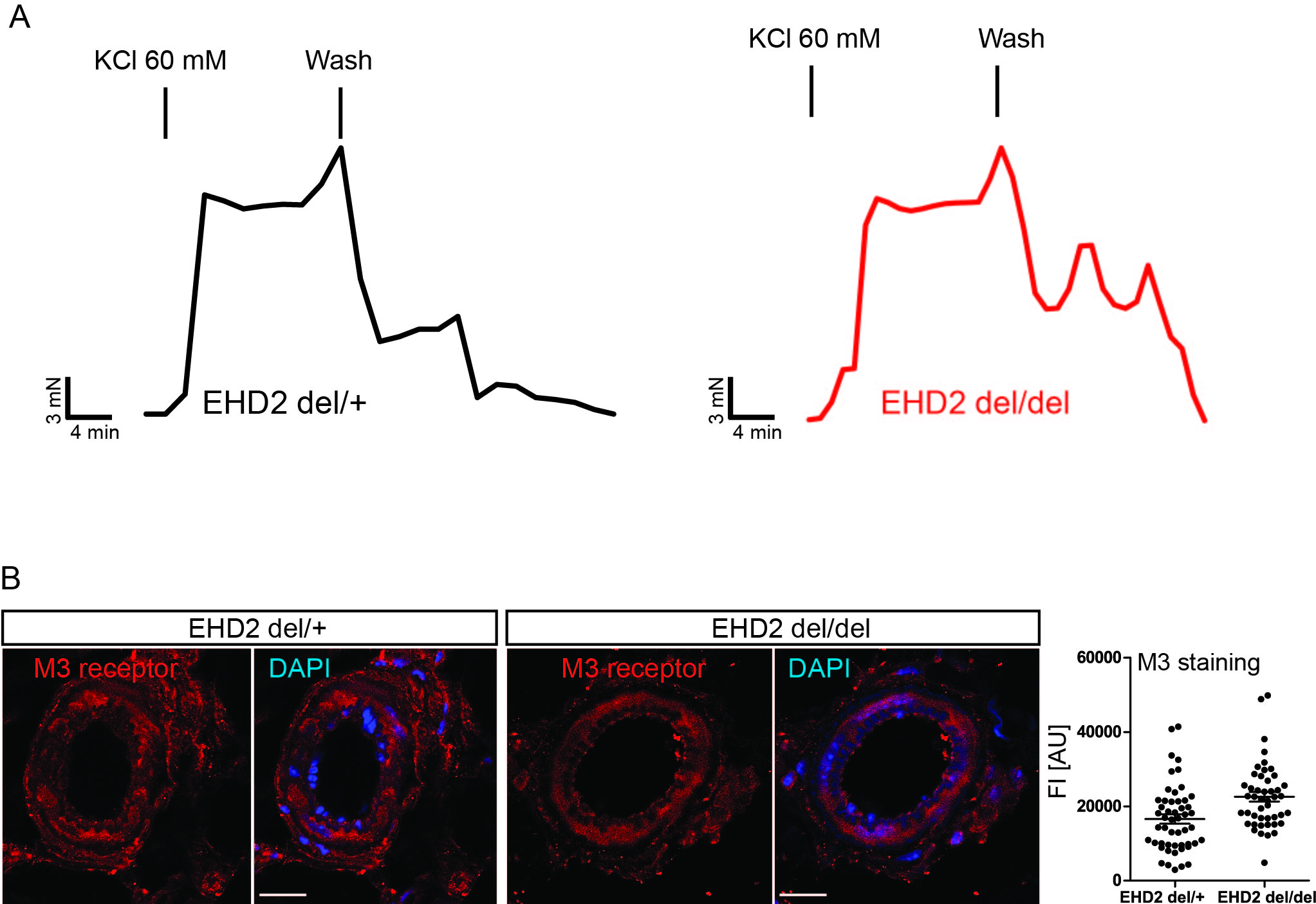

Supplement: S3 Fig — (TIF) [file pone.0223620.s004.tif]

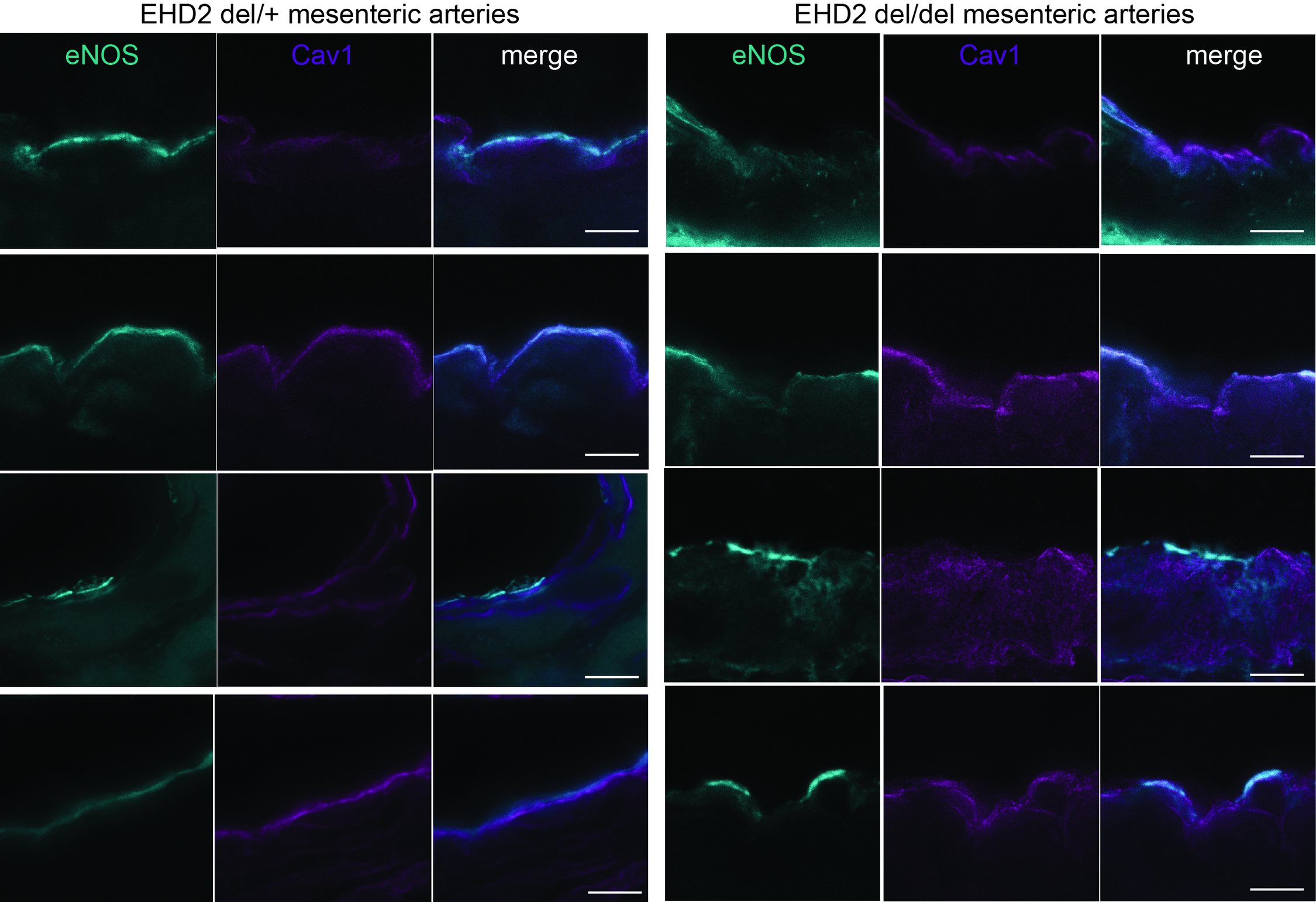

Supplement: S4 Fig — (TIF) [file pone.0223620.s005.tif]

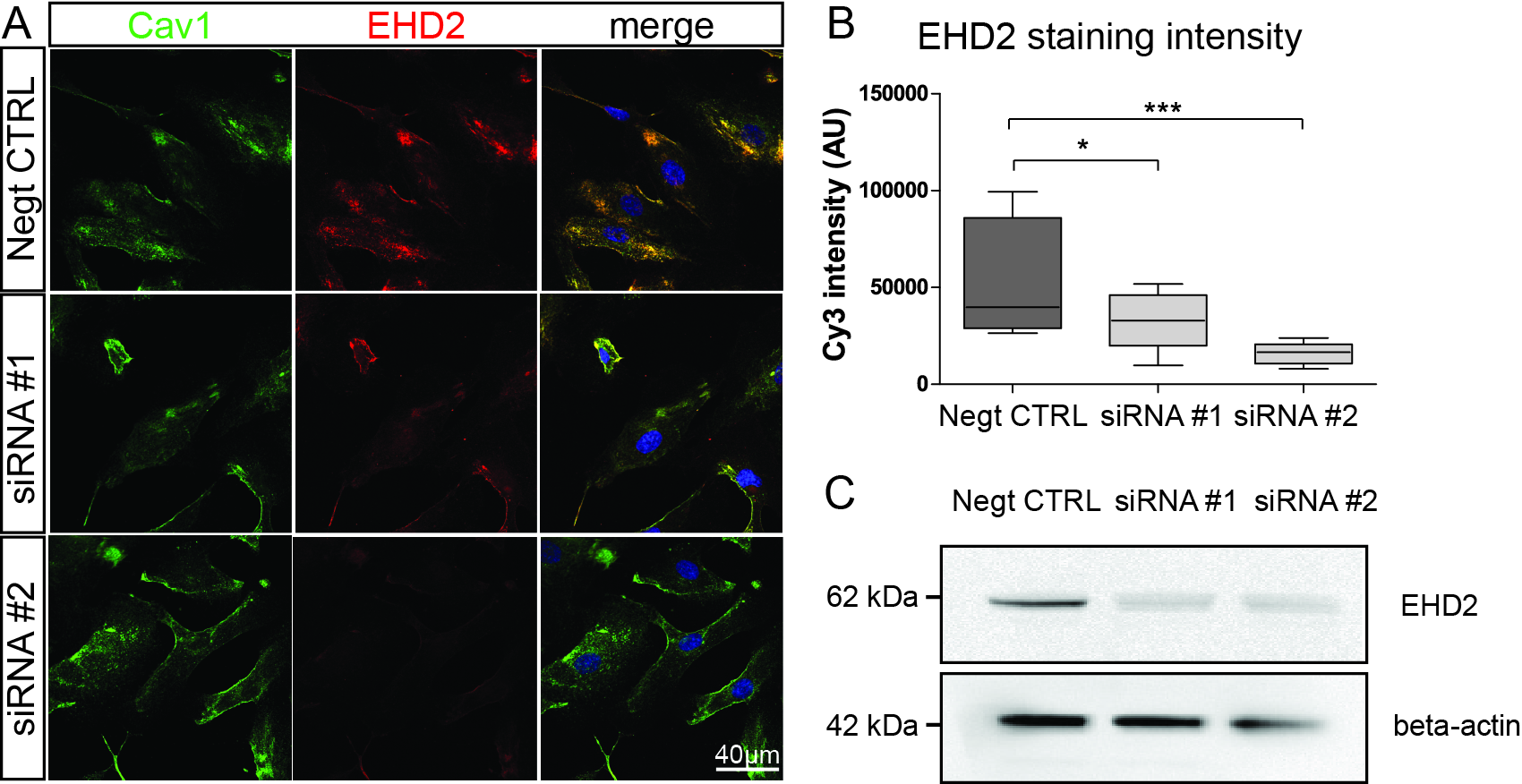

Supplement: S5 Fig — (TIF) [file pone.0223620.s006.tif]

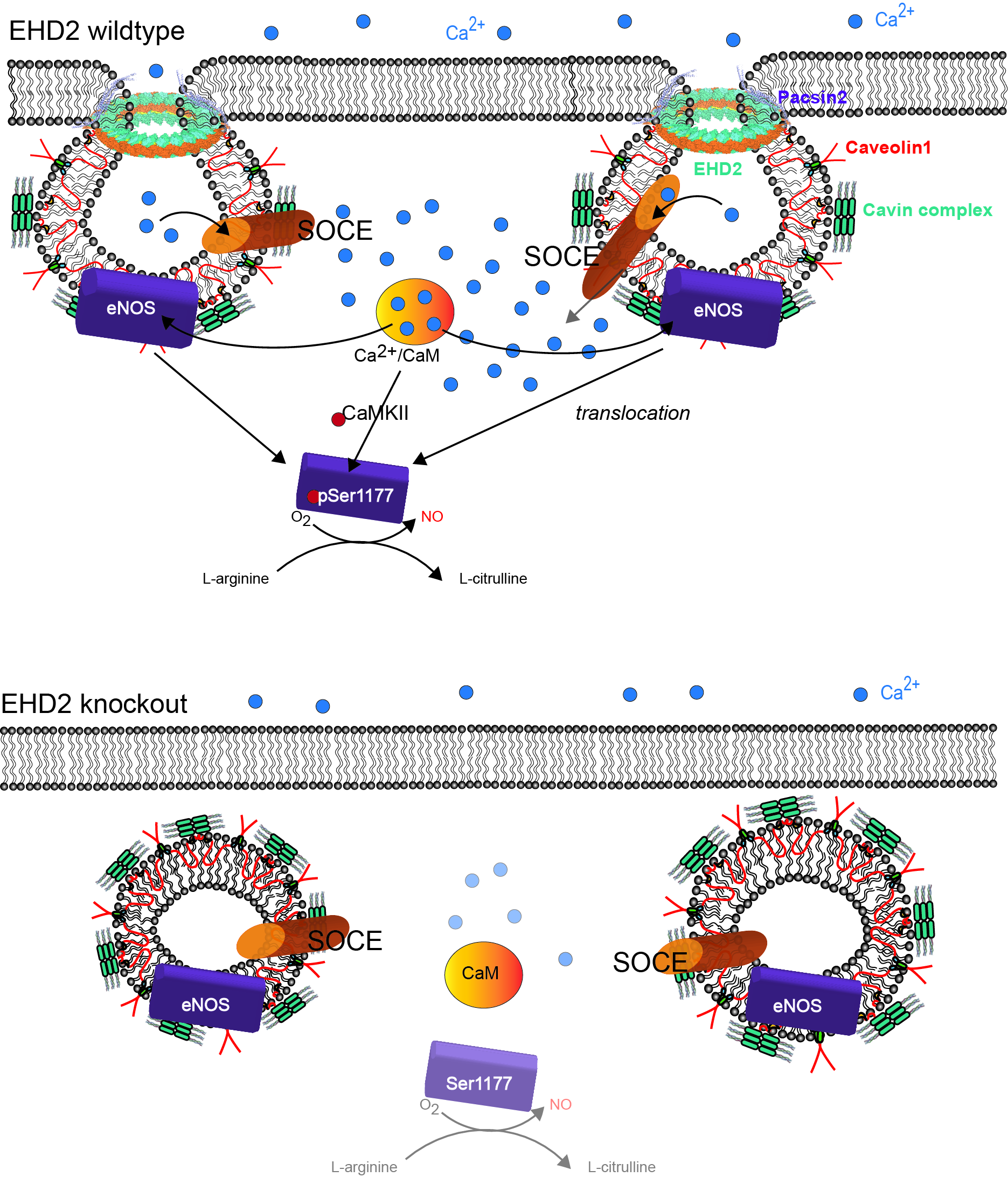

Supplement: S6 Fig — (TIF) [file pone.0223620.s007.tif]

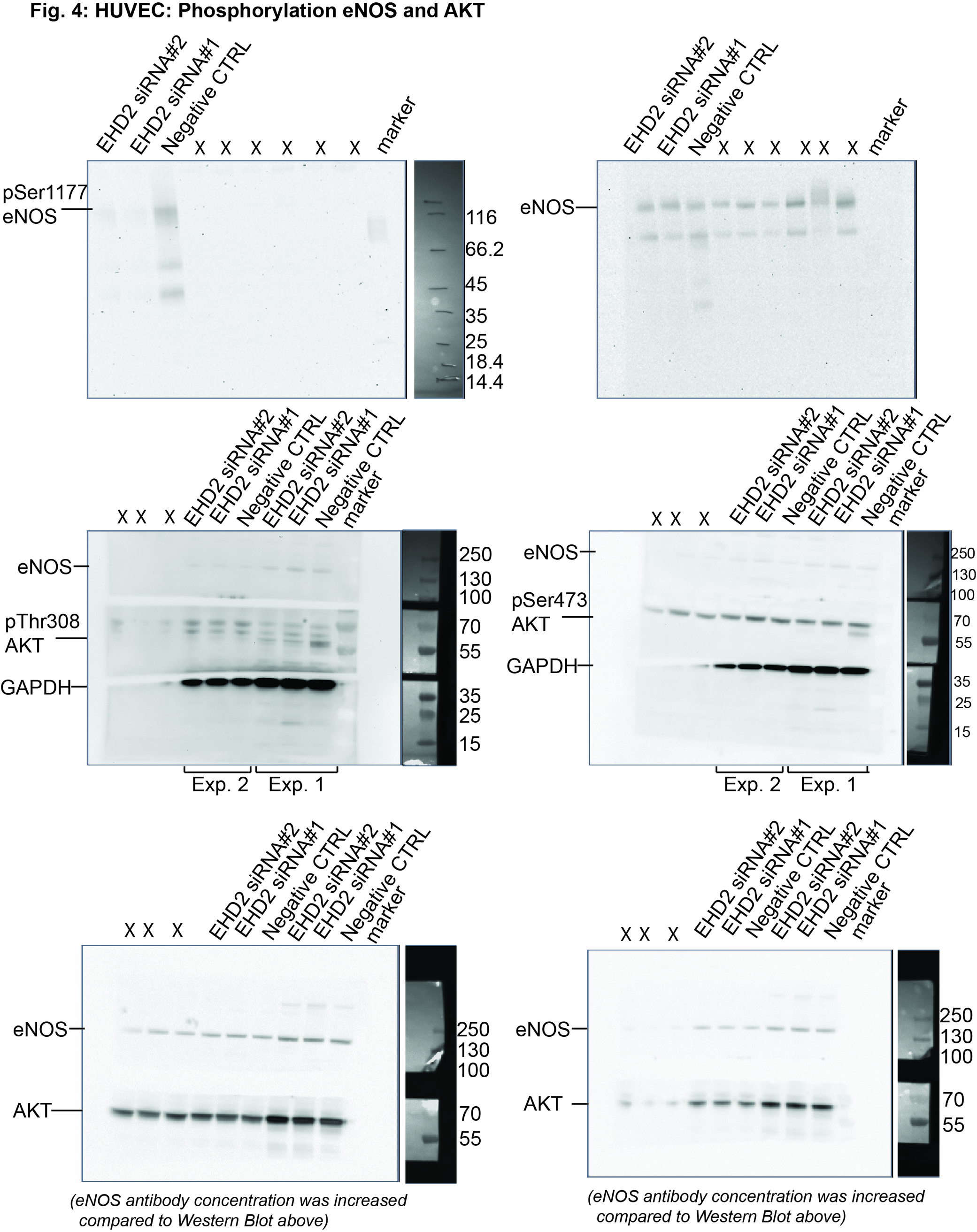

Supplement: S7 Fig — (TIF) [file pone.0223620.s008.tif]

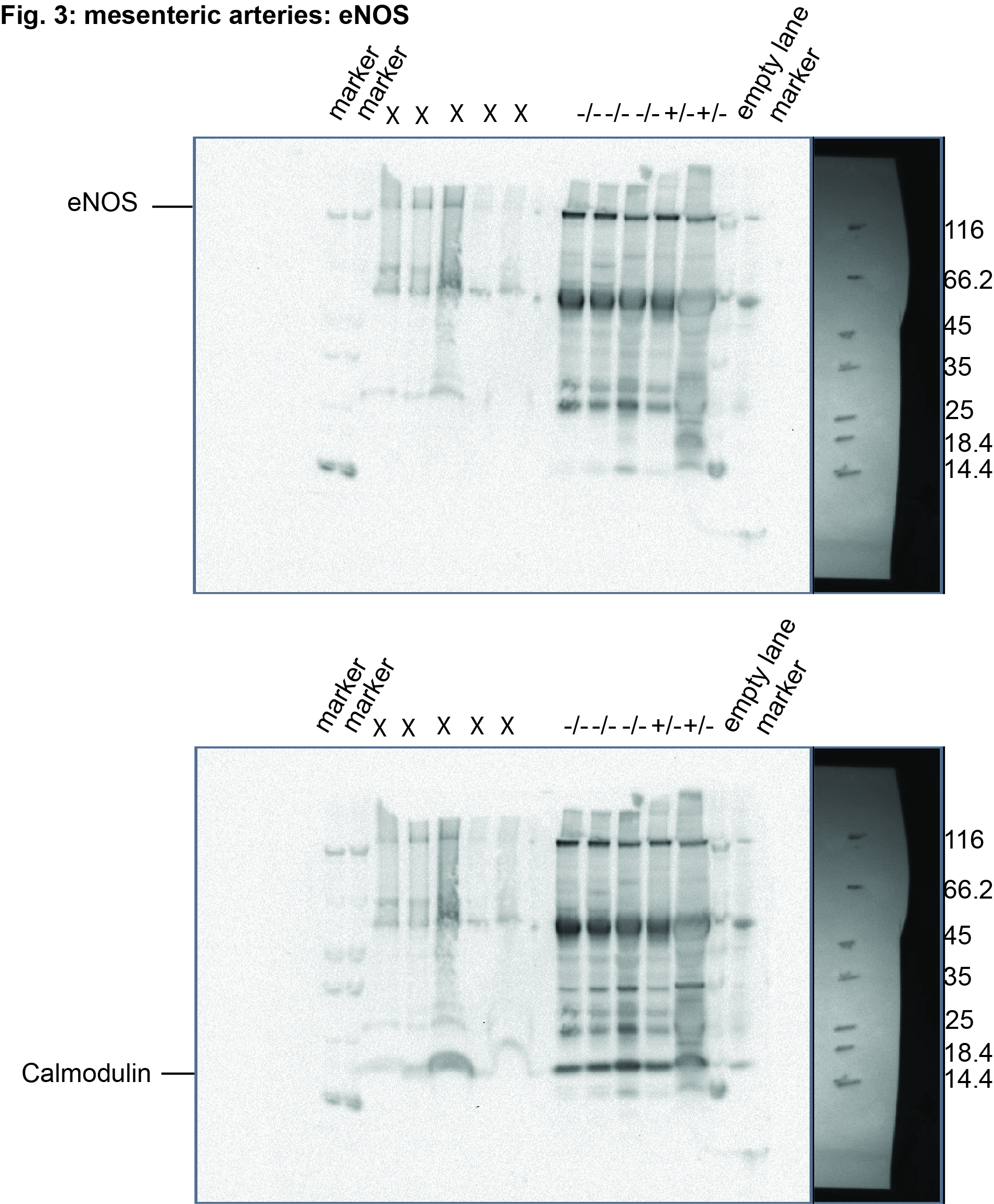

Supplement: S8 Fig — (TIF) [file pone.0223620.s009.tif]

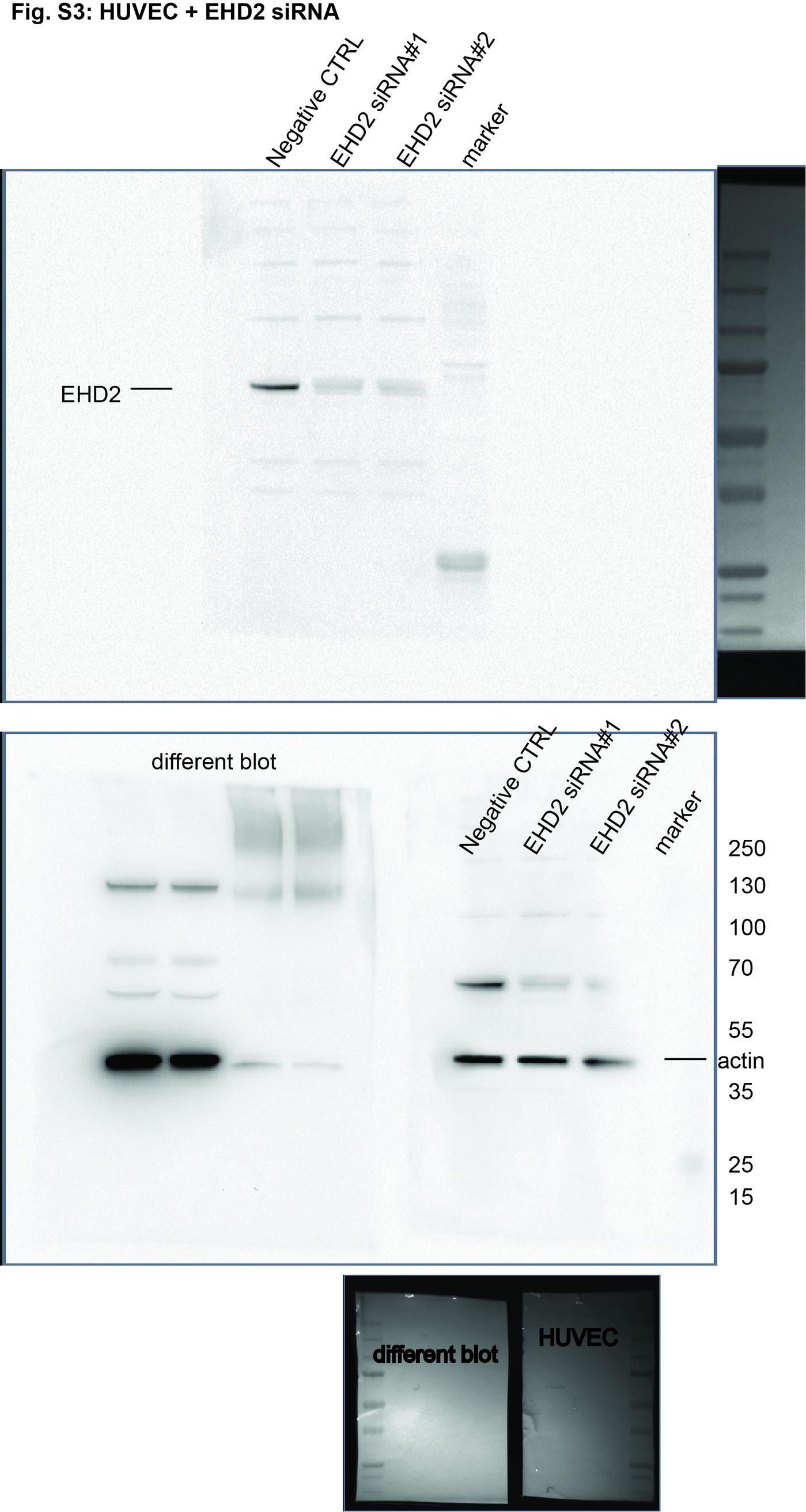

Supplement: S9 Fig — (TIF) [file pone.0223620.s010.tif]
